# Prospective association of occupational and leisure-time physical activity with orthostatic blood pressure changes in older adults

**DOI:** 10.1038/s41598-023-46947-7

**Published:** 2023-11-24

**Authors:** Agnieszka Kujawska, Sławomir Kujawski, Melanie Dani, Mitchell G. Miglis, David M. Hallman, Marat Fudim, Pinar Soysal, Jakub Husejko, Weronika Hajec, Natalia Skierkowska-Kruszyńska, Małgorzata Kwiatkowska, Julia L. Newton, Paweł Zalewski, Kornelia Kędziora-Kornatowska

**Affiliations:** 1 Department of Exercise Physiology and Functional Anatomy, Ludwik Rydygier Collegium Medicum in Bydgoszcz Nicolaus Copernicus University in Toruń, Świętojańska 20, 85-077 Bydgoszcz, Kujawsko-Pomorskie Poland; 2 Cutrale Peri-operative and Ageing Group, Imperial College London, London, W12 0BZ UK; 3 Department of Neurology and Neurological Sciences, Stanford University School of Medicine, Palo Alto, CA USA; 4 Department of Occupational Health Science and Psychology, University of Gävle, Gävle, Sweden; 5 Department of Medicine, Duke University Medical Center, Durham, NC USA; 6 Duke Clinical Research Institute, Durham, NC USA; 7 Institute of Heart Diseases, Wroclaw Medical University, Wroclaw, Poland; 8 Department of Geriatric Medicine, Faculty of Medicine, Bezmialem Vakif University, Istanbul, Turkey; 9 Department of Geriatrics, Collegium Medicum in Bydgoszcz, Nicolaus Copernicus University in Toruń, 85-094 Bydgoszcz, Poland; 10 Department of Basic Clinical Skills and Postgraduate Education of Nurses and Midwives, Faculty of Health Sciences, Collegium Medicum im. L. Rydygier in Bydgoszcz, Nicolaus Copernicus University in Toruń, Collegium Medicum in Bydgoszcz, 85-094 Bydgoszcz, Poland; 11 Department of Anesthesiology and Intensive Care, Professor Franciszek Łukaszczyk Oncology Center, 85-796 Bydgoszcz, Poland; 12 Population Health Sciences Institute, The Medical School, Newcastle University, Newcastle-Upon-Tyne, NE2 4AX UK; 13 Department of Experimental and Clinical Physiology, Laboratory of Centre for Preclinical Research, Warsaw Medical University, 1B Banacha Street, 02-097 Warsaw, Poland

**Keywords:** Neuro-vascular interactions, Stress and resilience, Hypertension, Risk factors, Epidemiology, Ageing

## Abstract

Orthostatic hypotension (OH) is common in older people. We examined the influence of self-reported occupational-related physical activity (PA) and leisure-time physical exercise (PE) on orthostatic response in a sample of older people over a 2 year period. Supine and orthostatic systolic blood pressure (sBP), diastolic blood pressure (dBP), and mean blood pressure (mBP) were assessed in response to Active Stand (AS) test in 205 older subjects (> 60 years old) at baseline and 2-year follow-up. OH was found in 24 subjects (11.71%) at baseline and 20 subjects (9.76%) after 2 years, with a significant degree of variability in the occurrence of OH after 2 years. Twenty-two subjects who had OH at baseline were free of it after 2 years, two subjects had persistent OH at baseline and after 2 years. After 2 years, adults with occupational PA showed no significant decrease of blood pressure in response to AS test, while lack of undertaking an occupation-related PA was significantly related with a greater decrease in sBP and mBP in response to AS testing in the 1st min. Occupation-related PA and leisure-time-related PE were related to an increase in the response of BP on AS in change between baseline and after 2 years. High between-subjects variance in OH over 2 years was noted. Occupations that involved continuous physical activity and leisure-time physical exercise in middle age were both protective for BP decline on orthostatic stress test within 2 years.

## Introduction

Orthostatic hypotension (OH), defined as a sustained fall of more than 20 mmHg systolic blood (sBP) pressure or 10 mmHg diastolic blood pressure (dBP) within three minutes of standing is common in older people, with a reported prevalence of up to 20% of those in the community and 25% of institutionalised older patients^1,2^. Neither benign nor incidental, it is associated with injurious falls, fear of falling, stroke, dementia, increased cardiac bad outcomes (higher risk of congestive heart failure and coronary heart disease), and all-cause mortality^3–8^. In a middle-aged sample, the presence of OH (especially a drop in dBP) was related to a greater risk of cardiovascular disorders^9^. In addition, the presence of OH in mid-life is associated with higher risks of carotid intimal thickness, and carotid plaque, myocardial infarction, congestive heart disease, coronary heart disease, and all-cause mortality, highlighting an important time window for modulation^10^.

On the other hand, it seems that manual workers with low cardiorespiratory fitness might have increased cardiovascular mortality if they undertake intensive (that might be excessive in comparison to their fitness level) occupational-related physical activity (PA)^11–13^. Authors conclude that high levels of occupational physical activity may not result in the same health benefits as leisure time physical activity^14^. This phenomenon was called “the health paradox of occupational and leisure-time PA”^15^.

A previous study examined sBP response on orthostatic stress test within 4 years in patients over the age of 50^16^. Authors assessed longitudinal relationships of certain bad outcomes (decrease in cognitive function, falls, syncope, disability, and mortality) with 8 patterns of blood pressure response on the test. The present study, however, assessed longitudinal within-subject changes over a 2 year period in OH occurrence based on changes in sBP and dBP in older adults. In addition, there is a substantial gap in the knowledge on how the history of occupational-related PA and leisure-time-related physical exercise (leisure-time PE) impacts upon the response of blood pressure (BP) to an orthostatic stress test in older people. In the current study, we examined the influence of self-reported occupational-related PA and leisure-time PE on orthostatic response in a sample of older people over a 2 year period.

## Methods

### Study recruitment

Figure S1 shows the study recruitment process. Participants were recruited through advertisements on television radio, at public health lectures at Collegium Medicum, at meetings of organizations affiliated with older people in Bydgoszcz, and in daycare centers for older people. These advertisements described the opportunity to participate in physical health testing of health status, body composition and behavior in people aged 60 years and over. Study volunteers were excluded from participation if they were under 60 years of age. The study was approved by the ethics committee of the Ludwik Rydygier Memorial Collegium Medicum in Bydgoszcz, Nicolaus Copernicus University, Torun (KB 340/2015). Written, informed consent was obtained from all participants. Participants were examined between November 2015 and February 2020. A total of 407 participants (95 men, 312 women) participated in the baseline series of assessments and 205 returned 2 years later for a second assessment (40 men, 165 women). Our previous paper describes the results of comparison between re-examined and subjects who dropped out from the study^17^. All research was performed in accordance with relevant guidelines/regulations and in accordance with the Declaration of Helsinki.

### Assessment methods

#### Physical examination: blood pressure measurement and orthostatic hypotension measurements

A BP assessment was done based on the 2013 European Society of Hypertension (ESH) and of the European Society of Cardiology (ESC) Practice Guidelines^18^. All examinations were completed in a doctor’s office. Heart rate (HR) was derived from auscultation of the heart with a stethoscope while apical pulse was auscultated and expressed as beats per minute (bpm)^19^. BP was consecutively assessed using a mercury sphygmomanometer on both upper arms after at least 5 min’ supine rest^20^. The mean values for each sBP and dBP reading from these two assessments were analysed. Based on sBP and dBP, mean blood pressure (mBP) was calculated. The pulse pressure (PP) was derived as a difference between sBP and dBP.

After the measurement of BP in the supine position, an AS test was conducted. BP measurement was repeated at the 1st and 3rd mins of the standing. The presence of OH was defined by a sustained drop in BP of ≥ 20 mmHg sBP and/or ≥ 10 mmHg dBP, or a decrease in sBP to < 90 mmHg within three minutes of standing was observed^1,21^. An additional binary variable was created based on the presence of a decrease of ≥ 20 mmHg in sBP and/or ≥ 10 mmHg in dBP after 1st min of AS.

#### Self-reported occupation-related physical activity and leisure-time-related physical exercise

To measure leisure-time PE, a history of past professional and/or amateur sports involving at least three training sessions per week for a period of longer than four years when the individuals were aged 30–60 years was recorded. The age period of 30 to 60 years was chosen to serve as a control factor for occupational-related PA, that might take place in a similar age range. The presence of leisure-time PE was coded as a binary variable (present vs not present). Two questions regarding occupation were asked. First, participants were asked to select the category of their occupation from the following options: white-collar worker (a person who performs professional service, desk, or administrative work), white-collar worker in a managerial position, owner of the craft/entrepreneur, military/policeman/other uniformed services, seller/employee of trade, a farmer in an individual farm, physical worker-qualified, unskilled worker. Next, the following question was asked: “was your occupation related to bouts of continuous physical exertion”. The question regarded any lifetime period. If a discrepancy was noted, e.g. participants worked as a farmer and did not consider the work to be related to continuous physical exertion, then additional verification was carried out. Eventually, the presence of occupational-related PA was coded as a binary variable (present, if occupation was related to bouts of continuous physical exertion vs not present).

#### Covariates

Socioeconomic questionnaire data on items regarding smoking, current physical activity (PA), years of education, and occupational-related PA and leisure-time PE were collected based on subjects’ recall. Utilizing a questionnaire that was previously detailed, the frequency of current physical activities (PA) was evaluated^17^. Questions included items on the frequency of the physical activities: short walks around the house, long walks, gymnastics, cycling, running/jogging, swimming, skiing, team games, sailing, horse riding, Nordic walking, tennis/table tennis, dancing, work on individuals’ allotment plots or in the garden/mushroom collection. Higher questionnaire scores on the current PA level, indicated more frequent physical activity undertaken. In addition, data regarding the number of years in education were collected. During the physical examination, a history of disorders was examined including the presence of: diabetes type 2, pulmonary disease, Parkinson’s disease, stroke, arrythmias, hospitalizations because of heart disease (all of these variables were recorded after 2 years). In addition, the number of antihypertensive agents taken in between two time points of the study (before and after 2 years) were recorded.

Weight and muscle mass in kg were measured using Tanita BC-545 body-fat analyser at both time points. Muscle mass was predicted in kilograms and incorporated into the analysis as an alternative to body mass index^22^.

The Montreal Cognitive Assessment (MoCA) scale was used to evaluate cognitive function^23^. The range of score is from 0 to 30 points, with higher scores indicating better overall cognitive function.

### Power analysis

The current study is a secondary analysis of a previously published one^17^. Overall, 407 subjects (95 males) were examined at baseline, with 205 returning 2 years later for a second assessment (40 males). In the current study, post-hoc power analysis was done using simr package for the applied linear mixed model to test the effect of occupational-related PA in interaction between time of the AS test (supine, 1st min, 3rd mins) × time (before vs after 2 years) with sBP as predicted outcome^17,24^. Number of simulations was set on 1000 and alpha = 0.05. Power of the study and its 95% confidence interval was calculated as 80.60% (78.01; 83.01), which could be considered as adequate.

### Statistical analysis

The R environment was used to conduct statistical analysis (R Core Team, 2020). Data on OH was missing from two subjects before and one subject after 2 years. It was decided to not handle the missing data in any way. To present the dynamics of the response of BP on the AS test, graphs were created using the ggplot2 package to reveal both changes of BP absolute values in mean and confidence interval of BP in response to the AS test as well as the course of data from individuals^25^.

Linear models were applied to assess the relationship between occupational-related PA and leisure-time PE and blood pressure response to the on AS test. In addition, an analysis of the change in AS response within two time points was adjusted for the following covariates (age, sex, muscle mass, mBP in supine, difference in PP baseline minus after 2 years, mean HR, smoking, number of antihypertensive drugs prescribed, presence of: diabetes type 2, pulmonary disease, Parkinson’s disease, stroke, arrythmias, hospitalizations because of heart disease, MoCA, years of education, current physical activity frequency). An analysis treating two-time points separately was performed (by analyzing predictors of AS from before and after 2 years using two separate databases). “Δ 1st min sBP”, “Δ 1st min dBP”, and “Δ 1st min mBP” indicate the difference between BP measured on 1st minute and supine in sBP, dBP, and mBP, respectively. “Δ 3rd min sBP”, “Δ 3rd min dBP”, and “Δ 3rd min mBP” indicate to the difference between BP measured at the 3rd min and supine in sBP, dBP, and mBP, respectively. AS response changes within 2 years is the difference between Δ of BP before and Δ of BP after 2 years. All linear models were created using the Lme4 package^26^. Satterthwaite's method for approximating degrees of freedom for the t-test was applied using LmerTest package^27^. Random effect included the subject factor, while the rest of the factors included were set as fixed. Package car was used to conduct type III ANOVA on the linear mixed models to test significance of interaction between occupation-related PA or leisure-time PE, time (before vs. after 2 years) and effects of AS test. Results of post-hoc tests were adjusted using Holm correction using lsmeans and multcomp packages. The confidence interval (95%) for the coefficient parameters was calculated. R^2^ value and its 95% confidence interval were calculated using r2glmm package. Plots illustrating the linear mixed models' results were created using the dotwhisker package^28^. Alluvial diagrams and frequency tables were created using Jamovi with the easyalluvial and ClinicoPath packages^29,30^. To compare participants who had a history of occupational-related PA vs those who had no occupational-related PA, an independent t-test was used if assumptions on normality and homogeneity of variance were met, otherwise, the Mann–Whitney *U* test was applied^31^.

## Results

### The course of orthostatic blood pressure changes over 2 years

In total, 205 subjects (40 males) were examined at both baseline and follow-up visits. The mean age of subjects re-examined after 2 years was 69.67 (CI 68.85; 70.5, range 60–88). Most subjects (84.39%) obtained full secondary education or higher. Table 1 shows demographic variables and confounders included in the linear mixed models. In total, 63 subjects with occupational-related PA in their middle age were characterized by a lower prevalence of women (22% vs 58.5% in a group with occupation not involving PA, *p* = 0.03) and lower prevalence of involvement in leisure time PE in their midlife (12% vs 39% in a group with occupation not involving PA, *p* = 0.02). In addition, participants with occupational-related PA spent fewer years on education (12.95 ± 3.2 years vs. 14.81 ± 3.5 years in a group with occupation not involving PA, *p* = 0.0001) and had lower MoCA scores, indicating worse cognitive function before and after 2 years (*p* = 0.0006 and *p* = 0.02, respectively).

**Table 1:** Variables are included in the linear mixed model.

Variable (unit)	Level of estimate	Mean ± SD/count (%)	Mean ± SD/count (%)	Mean ± SD/count (%)	*p*-value
		Whole sample (n = 205)	Occupational-related PA (n = 63)	Occ. NOT included PA (n = 142)	
Age (years)	Before	69.66 ± 6.0	69.79 ± 6.2	69.61 ± 5.9	0.83
Muscle mass (kg)	Before	45.54 ± 8.2	47.24 ± 9.1	44.77 ± 7.7	0.16
	After 2 years	44.87 ± 6.7	46.48 ± 8.3	44.18 ± 5.7	0.03
mBP in supine (mmHg)	Before	103.28 ± 12.5	103.92 ± 11.0	102.99 ± 13.2	0.32
	After 2 years	102.06 ± 12.7	99.94 ± 13.0	103 ± 12.5	0.07
PP diff baseline-after 2 years (mmHg)	after 2 years	1.95 ± 16.5	0.82 ± 18.3	2.45 ± 15.6	0.45
Mean HR (bpm)	Before	67.57 ± 8.0	66.78 ± 8.4	67.91 ± 7.7	0.29
	After 2 years	69.60 ± 9.8	69.17 ± 11.5	69.79 ± 9.0	0.4
Anti-HT drugs (no)	After 2 years	0.95 ± 0.08	1.02 ± 1.3	0.92 ± 1.1	0.86
MoCA [points]	Before	23.63 ± 3.5	22.41 ± 3.7	24.18 ± 3.3	0.0006
	After 2 years	23.21 ± 4.1	22.24 ± 4.2	23.65 ± 3.9	0.02
Education (years)	Before	14.37 ± 3.4	12.95 ± 3.2	14.81 ± 3.5	0.0001
PA frequency (points)	Before	20.06 ± 7.2	20.38 ± 7.4	19.92 ± 7.2	0.56
	After 2 years	18.56 ± 7.7	19.41 ± 8.5	18.18 ± 7.3	0.4
Sex	Male	40 (19.5%)	18 (8.8%)	22 (10.7%)	0.03
	Female	165 (80.5%)	45 (22%)	120 (58.5%)	
Current smoker	No	165 (80.5%)	54 (26.6%)	111 (54.7%)	0.33
	Yes	38 (18.5%)	9 (4.4%)	29 (14.3%)	
	Missing data	2 (1.0%)	1 (0.5%)	1 (0.5%)	
Diabetes type II (presence)	No	173 (84.4%)	51 (24.9%)	122 (59.5%)	0.37
	Yes	32 (15.6%)	12 (5.9%)	20 (9.8%)	
Pulmonary disease (presence)	No	190 (92.7%)	60 (29.3%)	130 (63.4%)	0.56
	Yes	15 (7.3%)	3 (1.5%)	12 (5.9%)	
PD (presence)	No	202 (98.5%)	61 (29.8%)	141 (68.8%)	0.22
	Yes	3 (1.5%)	2 (1%)	1 (0.5%)	
Stroke (presence)	No	198 (96.6%)	61 (29.8%)	137 (66.8%)	1.00
	Yes	7 (3.4%)	2 (1%)	5 (2.4%)	
Cardiac arrhythmias (presence)	No	188 (91.7%)	57 (27.8%)	131 (63.9%)	0.78
	Yes	17 (8.3%)	6 (2.9%)	11 (5.4%)	
Heart disease hospitalization (presence)	No	187 (91.7%)	58 (28.4%)	129 (63.2%)	0.60
	Yes	17 (8.3%)	4 (2%)	13 (6.4%)	
	Missing data	1 (0.5%)	0 (0%)	1 (0.5%)	
Leisure time PE program from 30 to 60 year old (presence)	No	100 (49%)	39 (19.1%)	61 (29.9%)	0.02
	Yes	104 (51%)	24 (11.8%)	80 (39.2%)	

*mBP* mean blood pressure, *PP* pulse pressure, *HR* heart rate, *Anti-HT drugs* antihypertensive drugs, *MoCA* Montreal Cognitive Assessment, *PA* physical activity, *PD* Parkinson’s Disease, *PE* physical exercise.

**Figure 1:**
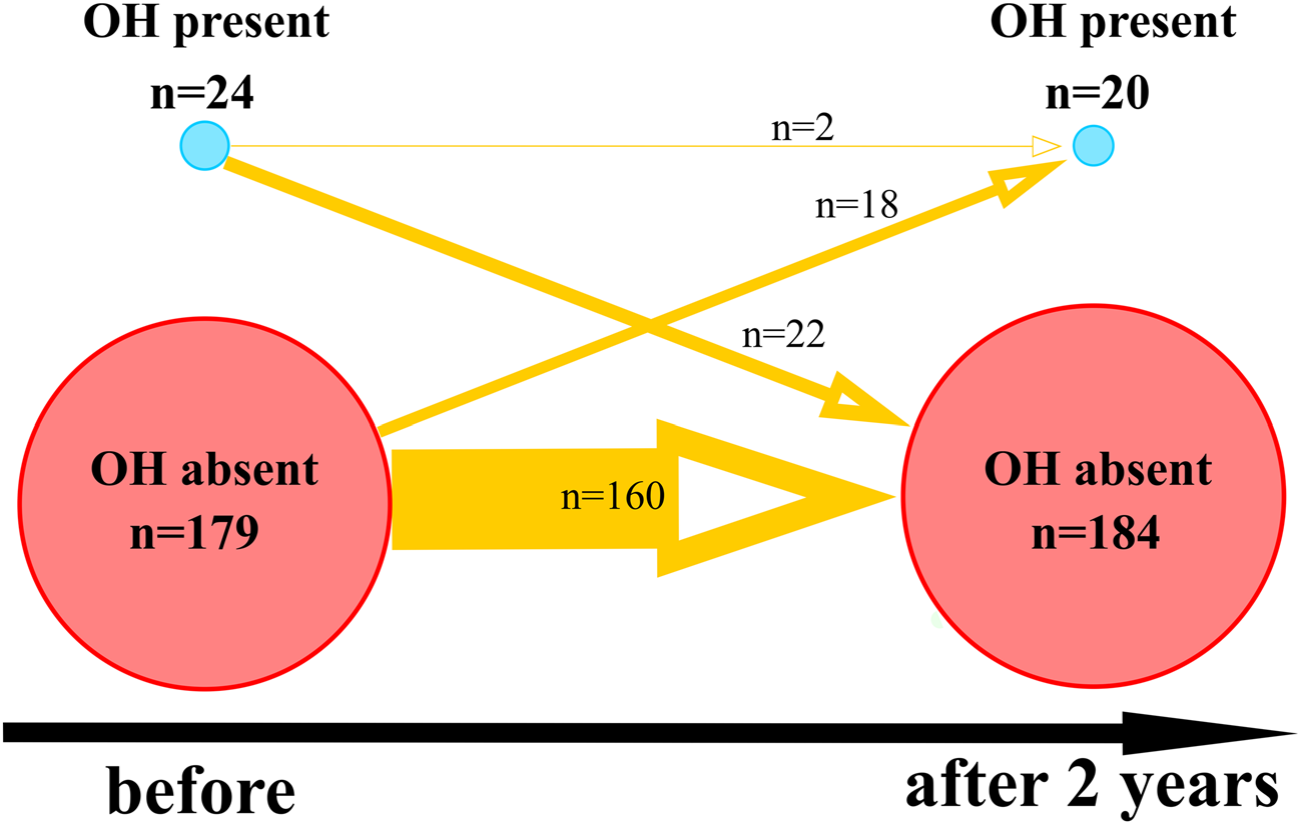
Network graph showing dynamics of OH presence before and after 2 years. The black horizontal arrow denotes the timeline. Blue dots denote the subgroup of subjects with OH present, and red dots denote the subgroup without OH. The size of the dots is proportional to the number of subjects in a specific category. Orange, directed arrows show change in time (baseline vs after 2 years) in the number of subjects in specific categories. The width of the orange arrows is proportional to the numbers on the orange arrows which denote the number of subjects that moved from a particular category after 2 years in comparison to the baseline. Missing data (n = 2 before and n = 1 after 2 years) is not shown to increase clarity of the figure.

Overall, sustained OH, was found in 24 subjects (12%) before and 20 subjects (10%) after 2 years (Fig. 1). Twenty-two (11%) participants who had OH at baseline were free of OH after 2 years (Fig. 1). Two (1%) subjects had persistent OH before and after 2 years (Fig. 1). Eighteen (9%) subjects were free of OH during baseline however developed OH after 2 years (Fig. 1).

Data on the response of BP on the AS test during the 1st minute of the AS test, 3rd min of AS before and after 2 years is presented in Table 2. Relatively high heterogeneity in response to AS testing was noted (SD ranging from 8.1 mmHg for dBP to 17.3 mmHg in sBP before and from 6.8 mmHg in mBP to 14.9 mmHg after 2 years) (Table 2).

**Table 2:** Response of BP on AS test during 1st min of AS test, 3rd min of AS before and after 2 years. Discrepancies between absolute and relative values are caused by missing data on BP response to the AS test (n = 2 before and n = 1 after 2 years).

AS test time-point	Before			After 2 years		
	sBP (mmHg) mean ± SD	dBP (mmHg) mean ± SD	mBP (mmHg) mean ± SD	sBP (mmHg) mean ± SD	dBP (mmHg) mean ± SD	mBP (mmHg) mean ± SD
Supine	140.26 ± 19	85.06 ± 10.7	103.28 ± 12.5	137.73 ± 18	84.49 ± 12.1	102.06 ± 12.7
1st min	135.53 ± 20.8	83.65 ± 12.4	100.77 ± 13.8	134.83 ± 19.2	84.01 ± 12.4	100.78 ± 13.4
3rd min	137.96 ± 19.5	84.19 ± 11.8	101.94 ± 13.5	136.07 ± 19.1	84.16 ± 12.8	101.29 ± 13.3
Δ 1st min–supine	− 4.75 ± 14.5	− 1.46 ± 7.5	− 2.55 ± 8.1	− 2.94 ± 12	− 0.47 ± 8.3	− 1.13 ± 8.2
Δ 3rd min–supine	− 2.37 ± 11.7	− 0.91 ± 6.8	− 1.39 ± 7.2	− 1.7 ± 10.3	− 0.33 ± 9.4	− 0.78 ± 8.2
Δ 3rd min–1st min	2.35 ± 12.9	0.52 ± 5.9	1.13 ± 6.9	1.24 ± 9.9	0.15 ± 7.2	0.51 ± 6.8

### Relationship of occupation-related physical activity and leisure-time-related physical exercise with the course of orthostatic blood pressure changes in older people

**Figure 2:**
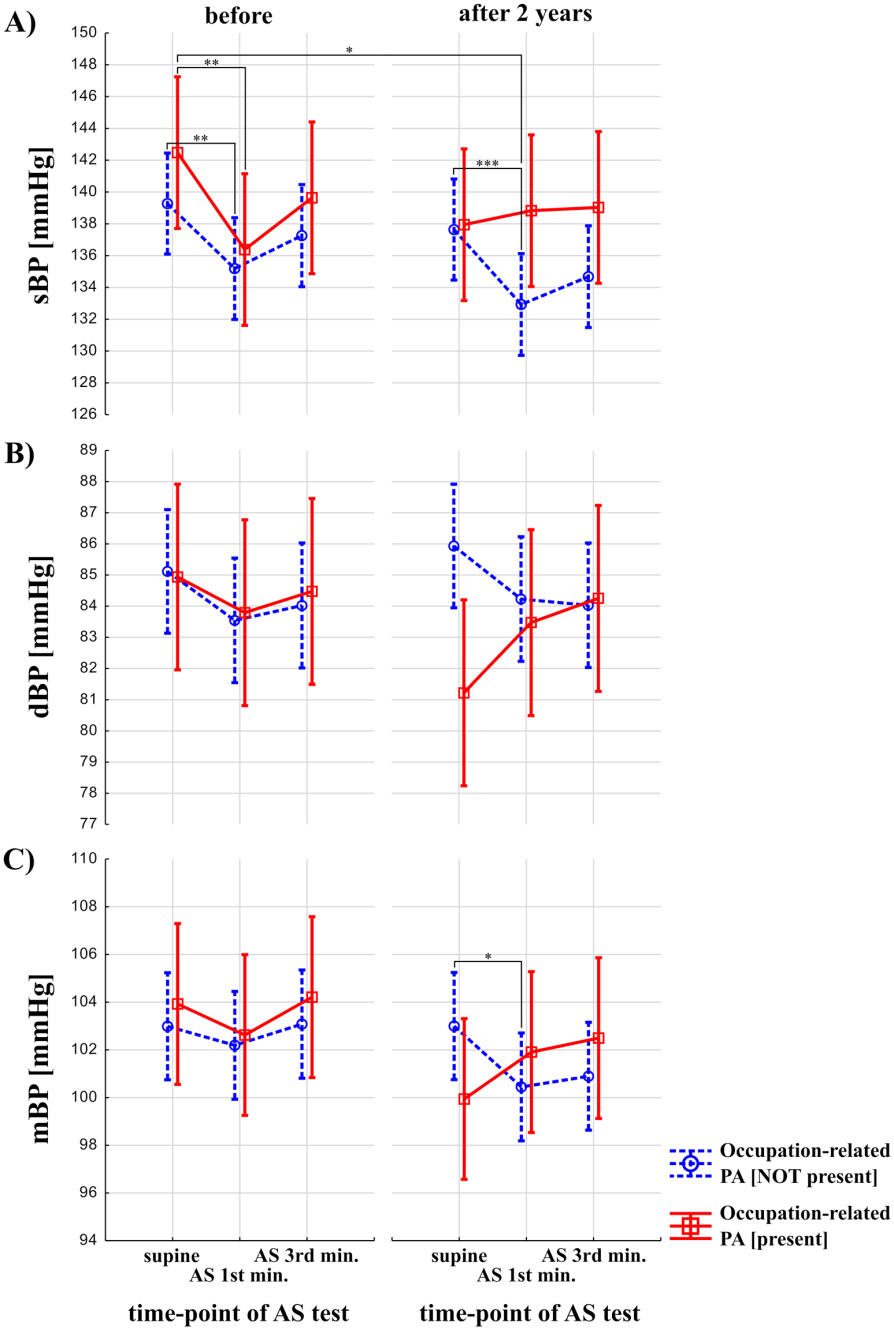
Blood pressure response to Active Stand (AS) test in older adults with and without a history of occupation-related physical activity (PA). The red, solid line indicates participants with occupational-related PA. The blue, dashed line represents participants without occupational-related PA. *p* < 0.0001 is denoted as "***", *p* < 0.001 as "**", *p* < 0.01 as "*".

Figure 2 presents changes (before vs after 2 years) in BP in response to AS testing between groups (participants with and without occupational-related PA). Effecst of occupation-related PA and time (before vs after 2 years) were statistically not-significant on sBP (F = 2.22, *p* = 0.14, and F = 1.04, *p* = 0.31, respectively), while effects of AS test and interaction between occupation-related PA, time and effects of AS test were statistically significant (F = 14.92, *p* < 0.0001, and F = 4.33, *p* = 0.01, respectively) (Fig. 2A). Post-hoc analysis revealed that in the group with occupation-related PA not present, sBP was significantly higher during rest vs in 1st minute after 2 years (137.64 ± 19.1 vs. 133.05 ± 18, *p* = 0.0003) (Fig. 2A). In the group without occupation-related PA in 1st minute of AS test after 2 years, sBP was significantly lower than in the group with occupation-related PA during rest at the baseline (133.05 ± 18 vs. 142.48 ± 19.1, *p* = 0.04) (Fig. 2A). In the group with and without occupation-related PA present, sBP was significantly higher during rest vs in 1st minute at the baseline (142.48 ± 19.1 vs. 136.38 ± 20.1, *p* = 0.004, and 139.27 ± 19.1 vs. 135.15 ± 21.2, *p* = 0.003, respectively) (Fig. 2A). The effects of occupation-related PA, AS test and time (before vs after 2 years) were statistically not significant on dBP (F = 0.45, p = 0.5, F = 1.03, *p* = 0.36, and F = 0.07, p = 0.79, respectively), while effects of interaction between occupation-related PA, time and effects of AS test was statistically significant (and F = 3.91, *p* = 0.02, respectively) (Fig. 2B). However, post-hoc analysis revealed no significant results. Effect of occupation-related PA, time (before vs after 2 years), and AS test were statistically not significant on mBP (F = 0.09, *p* = 0.77, F = 1.64, *p* = 0.20, F = 2.24, *p* = 0.10, respectively). Interaction between all three terms was statistically significant in the case of mBP (F = 4.52, *p* = 0.01). Results of post-hoc analysis showed that in the group without occupation-related PA present, mBP was significantly higher during rest vs in 1st minute after 2 years (103 ± 13.2 vs. 100.50 ± 15.8, *p* = 0.02) (Fig. 2C).

**Figure 3:**
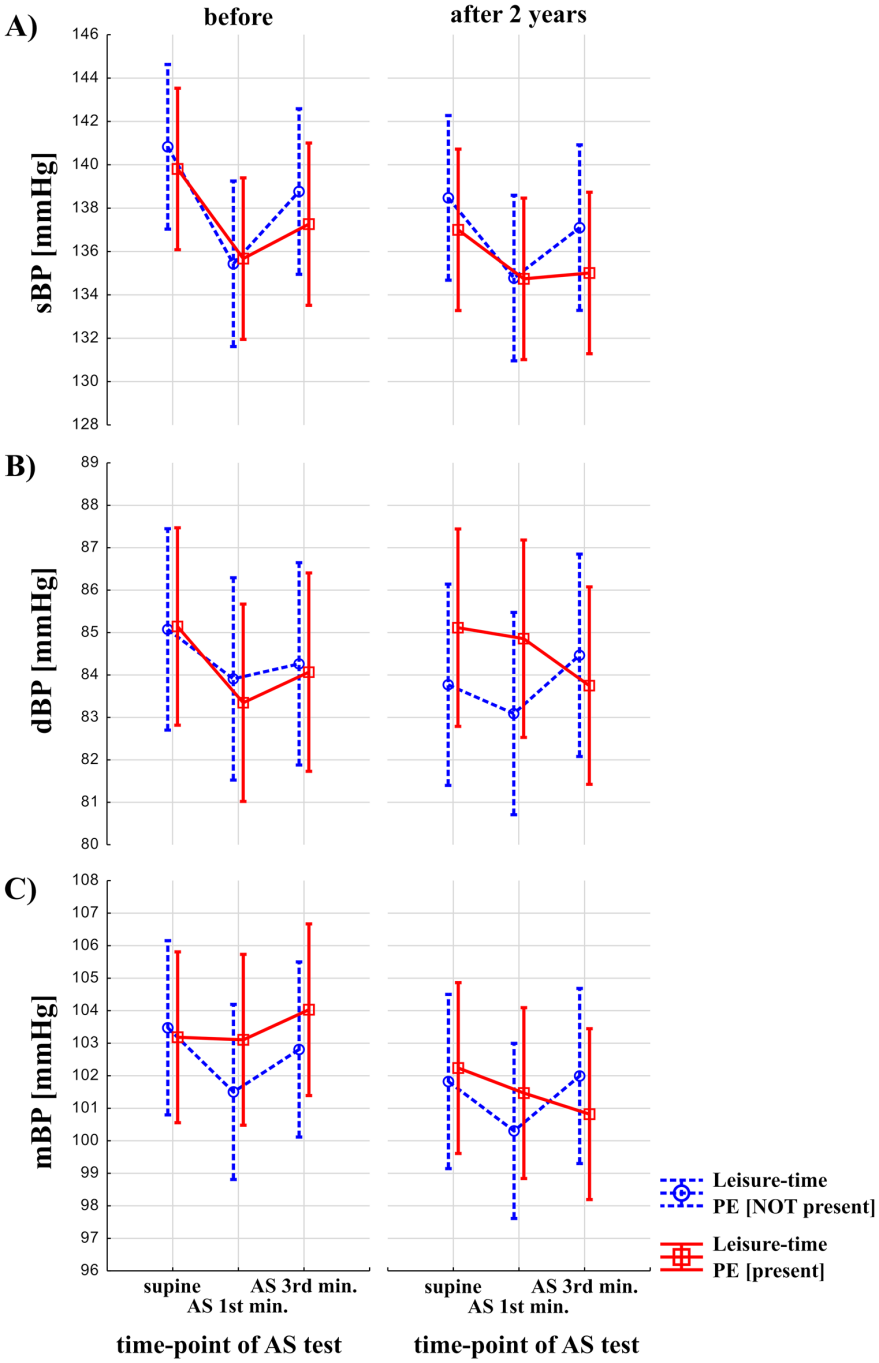
Blood pressure response to Active Stand (AS) test in older adults with and without a history of leisure-time physical exercise (PE). The red, solid line indicates participants with leisure-time PE. The blue, dashed line represents participants without leisure-time PE.

Results on the interaction between the effects of leisure-time PE, time, and effects of the AS test are shown on Fig. 3. Effects of leisure-time PE and time (before vs after 2 years) were statistically not significant on sBP, dBP and mBP. Effects of AS test were significant both for sBP, dBP, mBP (F = 21.32, p < 0.00001, F = 3.63, *p* = 0.03, F = 4.02, *p* = 0.02, respectively). Interaction between the presence of the leisure-time PE, time (before vs after 2 years) and AS test was not statistically significant (*p* > 0.05).

**Figure 4:**
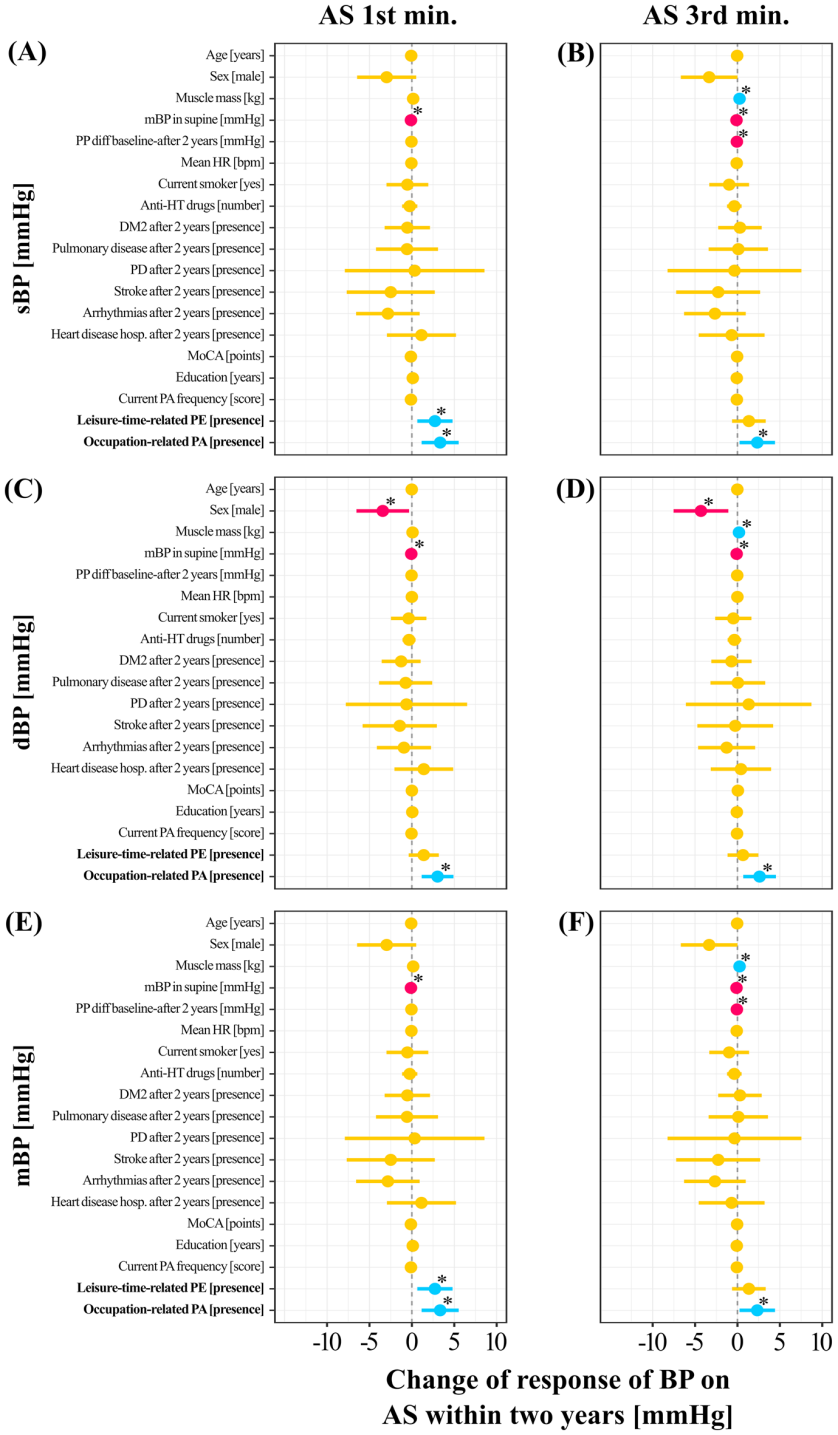
Mixed linear models predicting blood pressure changes. Dynamics of response of sBP on the AS test before vs after 2 years during (**a**) 1st and (**b**) 3rd min; change in response of dBP on the AS test before vs after 2 years during (**c**) 1st and (**d**) 3rd min; and change in response of mBP on the AS test before vs after 2 years during (**e**) 1st and (**f**) 3rd min. The “0” on the horizontal axis denotes no changes in the response of blood pressure on the AS test within 2 years (denoted by a vertical dashed line). Predictors with negative and positive estimates are placed on the left side and right side of the dashed line, respectively. Negative estimates are likely indicating a less favorable response on the AS test (i.e. decrease in blood pressure). Red and blue horizontal lines illustrate predictors in which both confidence interval values are lower and higher than 0, respectively. Orange dots and horizontal lines illustrate predictors in which confidence interval values of estimates cross zero. Blue and red dots denote estimates with positive and negative values. Results of presented models examining relationship between change of response of blood pressure (BP) on Active Standing (AS test within 2 years with leisure-time related physical exercise (PE) and occupation-related physical activity (PA) are adjusted for age, male sex, muscle mass, mean blood pressure (mBP) in supine, pulse pressure (PP) difference between before and after 2 years, mean heart rate (HR) in supine, being current smoker, number of antihypertensive drugs prescribed ([number]), presence of disorders ([presence]): diabetes mellitus type (DM2), pulmonary disease, Parkinson’s Disease (PD), stroke, arrhythmias, heart disease hospitalization, The Montreal Cognitive Assessment (MoCA) score, years of education and the current PA frequency. "*" mean statistically significant predictors (p < 0.05).

Figure 4 presents predictors of the changes in BP in response to the AS test before vs after 2 years. When adjusting for all other factors, undertaking a leisure-time PE was related to an increase in 1st min sBP of 2.72 mmHg, *p* = 0.01 (Fig. 4A) and 1st min mBP of 2.72 mmHg, *p* = 0.01 (Fig. 4E). Occupational-related PA was related to an increase in 1st min sBP of 2.34 mmHg, *p* = 0.03, 3.03 mmHg of 1st min dBP, *p* = 0.002 (Fig. 4A,C), and 3.33 mmHg of 1st min mBP, *p* = 0.003 (Fig. 4E). In addition, occupational-related PA was related to an increase in 3rd min sBP of 2.34 mmHg, *p* = 0.03 (Fig. 4B), 2.61 mmHg of 3rd min dBP, *p* = 0.01 (Fig. 4D), and 2.34 mmHg of 3rd min mBP, *p* = 0.03 (Fig. 4F).

## Discussion

In the current study, OH was found in 11.71% of older individuals at baseline and 9.76% from 205 participants after 2 years, with a tendency of OH diagnosis to change over time. A recent meta-analysis indicated an OH prevalence of 20% in community-dwelling older people and 25% in the long-term care setting^2^. It is possible that, the lower presence of OH in the current study could be explained by the application of mercury sphygmomanometers and the auscultatory method, leading to operator dependence. Alternatively, the study population may be fitter than other populations previously sampled, since they were a volunteer cohort recruited from non-healthcare community settings. The COPERNICUS study sample was recruited from the general population of older people in Poland via regional media. Hence, a higher disease burden, as in samples drawn from clinical settings, might be related to higher OH occurrence. In the current study, twenty-two subjects who had OH at baseline were free of it after 2 years, and two subjects had persistent OH at baseline and after 2 years. Eighteen subjects were free of OH at baseline however developed OH after 2 years. Multiple longitudinal studies have examined the longer-term effects of OH on health and illness outcomes^3–10^. Moloney et al. described results from The Irish Longitudinal Study on Ageing (TILDA), however, results of sBP only are provided^16^. However, this was the first study, to our knowledge, to examine longitudinal trends of sBP and dBP response to AS test over a 2-year period.

Older people seem to be particularly susceptible to OH due to physiological and pathological age-related changes. It might be related to a high prevalence of OH with aging, occurring in up to 30% of subjects older than 70 years^32,33^. For example, ageing is associated with a physiological change in baroreflex sensitivity, as well as reduced number, density and sensitivity of adrenoceptors in the heart and lower limb vasculature^34,35^. Additionally, ageing-related conditions such as deconditioning can exacerbate it^36^. Medications are commonly associated with OH, particularly antihypertensives and psychoactive medications^37^. However, in the current study number of antihypertensive drugs was not significantly related to BP response on AS test. Finally, common ageing-related conditions such as essential hypertension, synucleinopathy, and diabetes are closely associated with the development of OH, along with their respective treatments, therefore results from the above study were adjusted for the selected comorbidities. In line with results of the current study, previous studies showed that exercise, both endurance and strength, improve symptoms and modulate the baroreflex^38–40^. The baroreflex—the autonomic-mediated buffering in beat-by-beat blood pressure in response to physiological changes is a central part of the response to orthostatic stress in humans. Baroreceptor sensitivity seems to be lower in older subjects^41^. However, undergoing regular aerobic exercise might presumably reduce the magnitude of this reduction^41^.

In the current study occupations involving continuous physical exertion and leisure-time physical exercise undertaken in mid-life (at least 3 sessions per week for more than 4 years in total from 30 to 60 years of age) were both related to a lower BP decline in response to orthostatic stress test within 2 years. Other authors have noted that a greater neural control could not offset the effects of carotid vascular stiffening, resulting in dysfunction in response to BP decline upon standing in older subjects^42^. Based on the results of the current study, it might suggested that occupations involving continuous physical activity served as a protective factor for the baroreflex dysfunction. Nevertheless, future studies should put this hypothesis to the test. This might be in contrast to studies suggesting a Physical Activity Health Paradox^15^. This paradox is based on the observations that manual workers with low cardiorespiratory fitness might have increased cardiovascular mortality if they undertake high (that might be excessive in comparison to their fitness level) occupational-related physical activity^15^. In turn, physical activity during leisure time should not be neglected in industrial workers with moderate to high occupational-related physical activity, as the former might induce health benefits^15^.

In this study, OH was present at baseline both in subjects whose occupation involved continuous bouts of PA and those without. Despite a similar BP trend in response to the AS test at baseline, the orthostatic BP decline seems to diminish within the following 2 years in subjects reporting occupation-related PA. Therefore, PA undertaken in midlife seems to be associated with recovery from OH in older age, instead of prevention thereof. We find no easy explanation for this result. A longitudinal study on a cohort of 394 healthy subjects showed that subjectively measured physical activity level, in a sample with a mean age of 54 years, was related to objectively measured physical activity level in the same sample after 13 years follow-up^43^. Participants in the higher quartile of physical activity level, assessed with accelerometry, at baseline were more active 13 years later^43^. Moreover, a bidirectional, longitudinal relationship between physical function and PA was observed, in which reduction in physical function more consistently predicted a decrease in PA level^44^. In addition, retirement might be a time-point where modification of the level of multiple PAs occurs^45^. Unfortunately, the influence of retirement was not examined in the current study. In the future, longitudinal studies including larger sample sizes of older participants should also examine the change of behavior in time. Presumably, occupation-related physical activity might be characterized by repeated movements without sufficient recovery time^46,47^. Such physical activity with insufficient recovery may impose an elevated allostatic load^48^. On the other hand, it is established that physical exercise might play a protective role in baroreflex function reduction induced by ageing^40^. This positive adaptation's underlying mechanism might be related to higher blood vessel distensibility and improved signal transduction in barosensitive areas of the carotid sinus and aortic arch. In addition, improvement of integration in the brainstem cardiovascular centres has been suggested^49^. Cognitive stress leads to a reset of baroreflex and sympathetic activity to change it to a higher operating range (by actions of the hypothalamus and periaqueductal gray area)^50^. Resetting of arterial baroreflexes is also observed while undertaking physical activity bouts with an intensity-dependent manner^51^. Many years of aerobic exercise sessions modulate the operating point of the vascular sympathetic baroreflex by changing the gating of sympathetic bursts in middle-aged men^52^.

In the current study, it might be concluded that an increase in supine mBP within 2 years was related to a greater degree of decline of sBP, dBP and mBP in response to AS test both at 1st and 3rd minute. For an increase in PP whilst supine within 2 years, which is an indicator of an increase in aortic stiffness, there was a greater degree of decline of sBP and mBP in response to AS test at the 3rd minute after standing. Males tended to have a greater degree of decline of dBP in response to AS test at the 1st and 3rd minute. An increase in muscle mass within 2 years was related to sBP, dBP, and mBP increase in response to the AS test at the 3rd min. These factors are well established. For example, low muscle mass is associated with OH, and accordingly, low body mass index sarcopenia is a risk factor for OH^53,54^. Similarly, aortic stiffness (a consequence of hypertension) is associated with OH which may be due to altered baroreceptor function^55,56^. Conversely, strength training programs are associated with improvement in baroreceptor function^38^.

Presumably, some confounders might be related to the response of BP on AS and to each other. For example, subjects with a less active lifestyle might have a greater likelihood of developing high BP and obesity, which might prompt the intensification of antihypertensive therapy. Consistently, in the current study, an increase in supine mBP within 2 years was associated with a greater decline of sBP and mBP in response to AS test both at the 1st and 3rd min and dBP at the 3rd min. In our previous study, we have shown that frequencies of food product consumption in the currently-examined older subjects are interrelated^57^. It potentially shows how complex the pattern of behaviour is in the process leading to obesity development.

The current study has several limitations that were described in a previous paper, one of them being the lower prevalence of men in comparison to women in the examined sample^17^. Within-subject changes of orthostatic BP responses might change in older people in a period of circa 83 days^58^. Therefore, the observed result may be related not solely to occupational-related physical exertion but also to other factors or even might occur by chance. Authors of future studies on the dynamics of blood pressure might apply correction using the reliability information^59^. Assuming changes in ≥ 20 mmHg in sBP and/or ≥ 10 mmHg dBP as clinically relevant, then changes in BP observed in the current study should rather not be considered as such, despite being statistically significant^1,21^. In the current study subjects with occupational-related PA in their middle age were characterized by a lower prevalence of women (22% vs 58.5% in a group with occupation not involving PA). This distinction might possibly reveal cultural norms in Poland regarding gender stereotypes in relation to the type of undertaken occupation. As the analysis in the current study would not be meaningful because of a relatively small sample size of subgroups, further studies should assess whether sex differences in occupation and leisure physical activity influence on BP response to orthostatic stress and OH. Occupational-related PA and leisure-time PE were related to each other in the current study, namely participants who had occupational-related PA in their middle age were also less likely to be involved in leisure-time PE. This in turn might disrupt drawing clear conclusions from the relationship of those two predictors with a response of BP in orthostatic stress. The lack of continuous BP, HR, stroke volume, cardiac output, and total peripheral resistance measurements was a limitation. Because of the lack of a continuous BP study, we were not able to examine the initial BP response just after standing and therefore it was not possible to determine delayed BP recovery. Future studies should incorporate more sophisticated devices to allow continuous capture of more hemodynamic parameters and their dynamics in response to orthostatic stress. In addition, data on behaviour was relatively sparse, i.e. we measured the subjective frequency of the current physical activity level, and binary (yes/no) data was collected on the former physical activity level related to leisure time and occupation. This might be burdened by the recall bias, and lead to us missing potentially important data in regards to OH, such as the objective intensity of physical activity and rest of the features. Exposure to other types of stressors related to a particular occupation, should also be examined. The clinical significance of orthostatic hypotension is related to the possible induction of cerebral hypoperfusion, and subsequent syncope and falls^60^. However, in the current study, an objective assessment of co-occurring symptoms was not conducted. In addition, potential OH etiology was not assessed. Inappropriate response to orthostatic stress might be related to a disturbance in cerebral blood flow and objective symptoms^61,62^. Future studies should examine the effectiveness of particular pharmacological and non-pharmacological therapies in mitigating improper response of cerebral blood flow on orthostatic stress in older people. Changes of vulnerability to an acute decrease in cerebral blood flow should be examined in a longitudinal manner.

## Conclusions

Occupation involving continuous physical activity and leisure-time physical exercise undertaken in middle age (at least 3 sessions per week for more than 4 years in total from 30 to 60 years old) were both related to lower BP decline on orthostatic stress test within 2 years. Despite familiar BP trends in response to AS test at baseline, the orthostatic BP decline seems to diminish within the following 2 years in participants reporting occupation-related PA. This provides essential information for counselling subjects during daily clinical practice and long-term risk stratification. Based on the results of the current study, it might be suggested that previous occupations involving continuous physical activity served as a protective factor against dysregulation of BP regulation in response to orthostatic stress. Nevertheless, future studies should put this hypothesis to the test.

### Supplementary Information

Supplementary Figure S1.

## Data Availability

Data will be made available on request send via email to S. Kujawski (e-mail, skujawski@cm.umk.pl).
