# Supplementary figures and images for: Prospective association of occupational and leisure-time physical activity with orthostatic blood pressure changes in older adults
Source: Sci Rep. 2023 Nov 24;13:20704. doi: 10.1038/s41598-023-46947-7 (PMC10673924; doi:10.1038/s41598-023-46947-7)

**
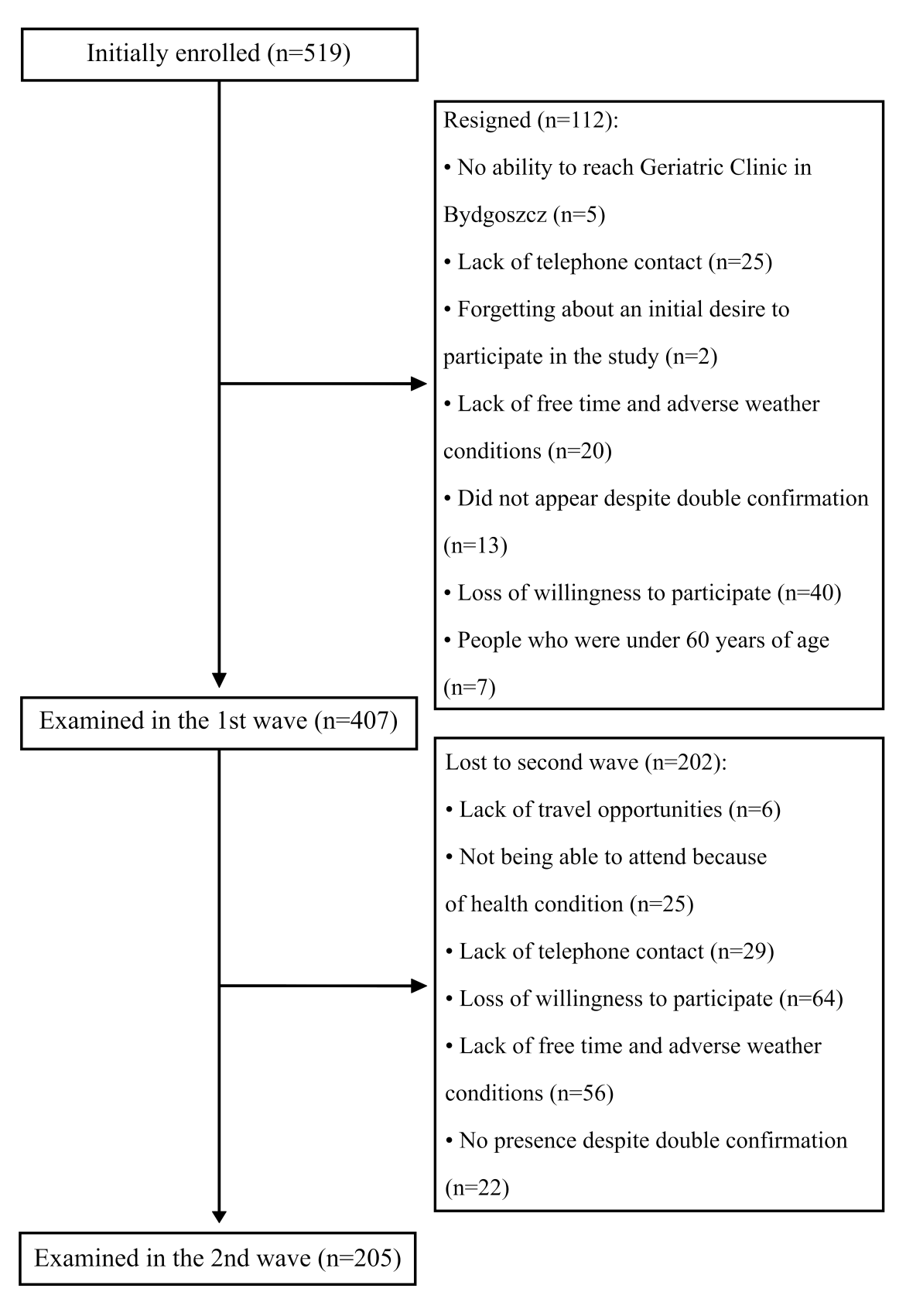
**

**Figure S1.** Flow chart of the study

Supplement: Supplementary file 1 — Supplementary Figure S1. [file 41598_2023_46947_MOESM1_ESM.docx]
